# Roles of TLR3 and RIG-I in Mediating the Inflammatory Response in Mouse Microglia following Japanese Encephalitis Virus Infection

**DOI:** 10.1155/2014/787023

**Published:** 2014-07-03

**Authors:** Rong Jiang, Jing Ye, Bibo Zhu, Yunfeng Song, Huanchun Chen, Shengbo Cao

**Affiliations:** ^1^State Key Laboratory of Agricultural Microbiology, Huazhong Agricultural University, Wuhan, Hubei 430070, China; ^2^College of Veterinary Medicine, Huazhong Agricultural University, Wuhan, Hubei 430070, China

## Abstract

Japanese encephalitis virus (JEV) infection can cause central nervous system disease with irreversible neurological damage in humans and animals. Evidence suggests that overactivation of microglia leads to greatly increased neuronal damage during JEV infection. However, the mechanism by which JEV induces the activation of microglia remains unclear. Toll-like receptor 3 (TLR3) and retinoic acid-inducible gene I (RIG-I) can recognize double-stranded RNA, and their downstream signaling results in production of proinflammatory mediators. In this study, we investigated the roles of TLR3 and RIG-I in the inflammatory response caused by JEV infection in the mouse microglial cell line. JEV infection induced the expression of TLR3 and RIG-I and the activation of extracellular signal-regulated kinase (ERK) and p38 mitogen-activated protein kinase (p38MAPK). Knockdown of TLR3 and RIG-I attenuated activation of ERK, p38MAPK, activator protein 1 (AP-1), and nuclear factor *κ*B (NF-*κ*B). Secretion of TNF-*α*, IL-6, and CCL-2, which was induced by JEV, was reduced by TLR3 and RIG-I knockdown and inhibitors of phosphorylated ERK and p38MAPK. Furthermore, viral proliferation was increased following knockdown of TLR3 and RIG-I. Our findings suggest that the signaling pathways of TLR3 and RIG-I play important roles in the JEV-induced inflammatory response of microglia.

## 1. Introduction

Japanese encephalitis, one of the leading forms of endemic encephalitis in Eastern and Southern Asia, is caused by Japanese encephalitis virus (JEV), which belongs to the family* Flaviviridae* [1]. Approximately 45,000 cases of the disease are reported annually, resulting in 10,000 to 15,000 deaths [2]. JEV infection primarily occurs in children. The clinical symptoms include headache, fever, vomiting, diarrhea, reduced levels of consciousness, and signs of meningeal irritation with polymorphic and diffuse pathological changes involving various parts of the nervous system [2, 3]. Patients infected with JEV characteristically experience persistent motor defects and severe cognitive and language impairments [4].

JEV targets the central nervous system (CNS), leading to neuroinflammation with typical features of immune cell infiltration, neuronal death, and the activation of resident glial cells. Various mechanisms including virus-mediated killing and cytokine-mediated cytotoxicity have been reported as the causes of neuronal death. Microglia comprise the resident mononuclear phagocytic population in the CNS parenchyma and represent an important component of the innate immune response against invading pathogens [5, 6]. Uncontrolled overactivation of microglia may play an important role in inducing neuron death owing to the production of proinflammatory mediators [2]. These factors, including inducible nitric oxide synthase, cyclooxygenase-2, interleukin- (IL-) 6, IL-1*β*, tumor necrosis factor- (TNF-) *α*, chemokine (C-C motif) ligand 2 (CCL2), and CCL5, are significantly increased in microglia following JEV infection [7]. The cross-talk between neurons and microglia often influences the outcome of JEV pathogenesis [8].

Activation of various pattern recognition receptors (PRRs), such as toll-like receptors (TLRs) and retinoic acid-inducible gene 1- (RIG-I-) like receptors, provides the first line of defense in the antiviral immune response by inducing the release of cytokines and chemokines [9]. TLR3 and RIG-I recognize double-stranded RNA (dsRNA) in innate immune cells during viral replication [9, 10]. The interaction of viral RNA with TLR3 and RIG-I may trigger several intracellular signaling pathways leading to activation of mitogen-activated protein kinases (MAPKs), extracellular signal-regulated kinase (ERK), p38, and c-Jun N-terminal kinase and culminate in the activation of nuclear factor *κ*B (NF-*κ*B) and induction of the expression of proinflammatory cytokines [11]. TLR3 and RIG-1 are widely expressed in the cells of the innate immune system, but they induce distinct cellular responses depending on the cell type [6]. TLR3 is responsible for the secretion of cytokines such as TNF-*α*, IL-12p40, and IL-6 in primary murine microglia following stimulation with poly(I : C) [12]. TLR3 upregulation has been observed in experimental simian immunodeficiency virus (SIV) infection and HIV encephalitis in humans, suggesting that enhanced TLR3 expression during viral infection may predispose cells to a greater response [13]. RIG-I activates p38MAPK, leading to secretion of C-X-C motif chemokine 10 (CXCL-10) and IL-12 in bone marrow-derived dendritic cells following viral infection [14]. RIG-I also recognizes JEV dsRNA and activates p38MAPK and NF-*κ*B signaling in various cells including neurons [8].

Microglia express a repertoire of PRRs. However, the mechanism by which JEV induces the activation of microglia remains unknown. In this study, we investigated the roles of TLR3, RIG-I, and the adaptor molecules involved in the activation of signaling for regulating the inflammatory response of microglia following JEV infection.

## 2. Methods

### 2.1. Virus and Cells

The mouse microglia cell line BV-2 was grown in Dulbecco's modified Eagle medium (DMEM) supplemented with 10% fetal bovine serum (FBS, GIBCO) and 1% penicillin/streptomycin (Sigma) at 37°C. JEV strain P3 was propagated in BHK-21 cells, and the viral titer was measured with a plaque assay [15].

### 2.2. Virus Infection

After incubation for 24 hours (h), the BV-2 cells were switched to serum-free medium for 12** **h and then adsorbed with JEV at a multiplicity of infection (MOI) of 0.01 or 1 for 1** **h (MOI = 0.01 for the viral proliferation assay, or MOI = 1 for determining cytokines and chemokines and signaling kinase expression). After adsorption, unbound viruses were removed by gentle washing with phosphate-buffered saline (PBS). Fresh medium was added to each well for further incubation at 37°C.

### 2.3. RNA Interference

Short hairpin RNAs (shRNAs) targeting TLR3 and RIG-I, negative control shRNA (CTR) targeting an irrelevant sequence, and primers used for qPCR of TLR3 and RIG-I expression were purchased from Genecopoeia. BV-2 cells were cultured at 1.5  × 10^5^ cells/well in 12-well plates and then transfected with RIG-I and TLR3 shRNA at 1.6**** *μ*g/well with X-tremeGENE HP DNA Transfection Reagent (Roche, Inc.). After incubation for 48** **h, cells were infected with JEV.

### 2.4. Inhibitor Treatment

The concentrations of inhibitors were selected and used as described [16, 17]. Briefly, inhibitors U0126 (Sigma-Aldrich) (10 *μ*M) and SB302580 (Sigma-Aldrich) (10 *μ*M), respectively, against phosphorylated ERK and p38MAPK were added to the medium immediately after 1 h of viral adsorption and continuously incubated during the experimental periods.

### 2.5. Immunofluorescence Assay

At 48 h postinfection (hpi), cells were fixed with methanol for 10 min and then permeabilized with 0.1% (w/v) Triton X-100 in PBS for 2 min on ice before incubating with 1% bovine serum albumin in PBS for 45 min at room temperature. After incubation with 10 mg/mL of a monoclonal antibody against JEV E protein for 2 h at room temperature, cells were incubated with a 1 : 300 dilution of Alexa Fluor 555-conjugated goat anti-mouse IgG (Invitrogen) for 45 min at room temperature before observation under a fluorescence microscope.

### 2.6. Quantitative Reverse Transcription-PCR (qRT-PCR)

Total cellular RNA was isolated using TRIzol reagent (Invitrogen) and reverse transcribed using a ReverTra Ace-*α* kit (TOYOBO). Levels of viral RNA and cytokine mRNA were determined with qRT-PCR using SYBR Green Real-Time PCR Master Mix (TOYOBO). Reactions were carried out on a StepOne Plus thermal cycler (Applied Biosystems). Specific forward and reverse primers for the JEV C gene and inflammatory cytokine genes are shown in Table 1. The thermal cycling program was 95°C for 10 min, followed by 40 cycles of 95°C for 15 s and 60°C for 30 s, with a final step of 72°C for 30 s. Moreover, a plasmid, pcDNA3.0-HA-C, was used to construct a standard curve to quantify the viral load in 10-fold dilutions with an initial concentration of 4 × 10^14^ copies/mL. The levels of cytokine mRNAs were normalized to the levels of the mouse housekeeping gene *β*-actin. The relative difference was calculated as the fold change using the equation 2^−ΔΔCt^.

### 2.7. Enzyme-Linked Immunosorbent Assay (ELISA)

Commercially available ELISA kits (Ebioscience) were used to quantify TNF-*α*, IL-6, and CCL-2 in the supernatants of BV-2 cells.

### 2.8. Western Blotting

Cells were washed twice with PBS and harvested in RIPA buffer. The nuclear proteins were extracted using NE-PER(R) Nuclear and Cytoplasmic Extraction kit (Thermo). Total cellular or nuclear extracts were separated with SDS-PAGE and electrophoretically transferred to a polyvinylidene difluoride membrane. After blocking with 5% nonfat milk in Tris-buffered saline for 1 h at room temperature, the membrane was incubated with primary antibodies against AP-1 (Santa Cruz Biotechnology), RIG-I, TLR3, NF-*κ*B, ERK, phospho-ERK, p38MAPK, and phospho-38MAPK (Cell Signaling Technology). After washing with Tris-buffered saline containing 0.5% (w/v) Tween [18], the membrane was incubated with an appropriate peroxidase-conjugated secondary antibody (Boster). Each blot was developed using SuperSignal West Pico and SuperSignal West Femto (Thermo). The images were captured using the MF-Chemi Bio Imaging System (DNR). To correct the results for potential variations in sample load, each blot was stripped and reprobed with anti-*β*-tubulin or anti-GAPDH (Santa Cruz Biotechnology). The fold changes of protein bands were analyzed with Image J software.

### 2.9. Statistical Analysis

Data between groups were compared using one-way analysis of variance. Statistical significance was set at *P* < 0.05 for all analyses.

## 3. Results

### 3.1. Expression of RIG-I and TLR3 Is Upregulated in BV-2 Cells following JEV Infection

**Figure 1:**
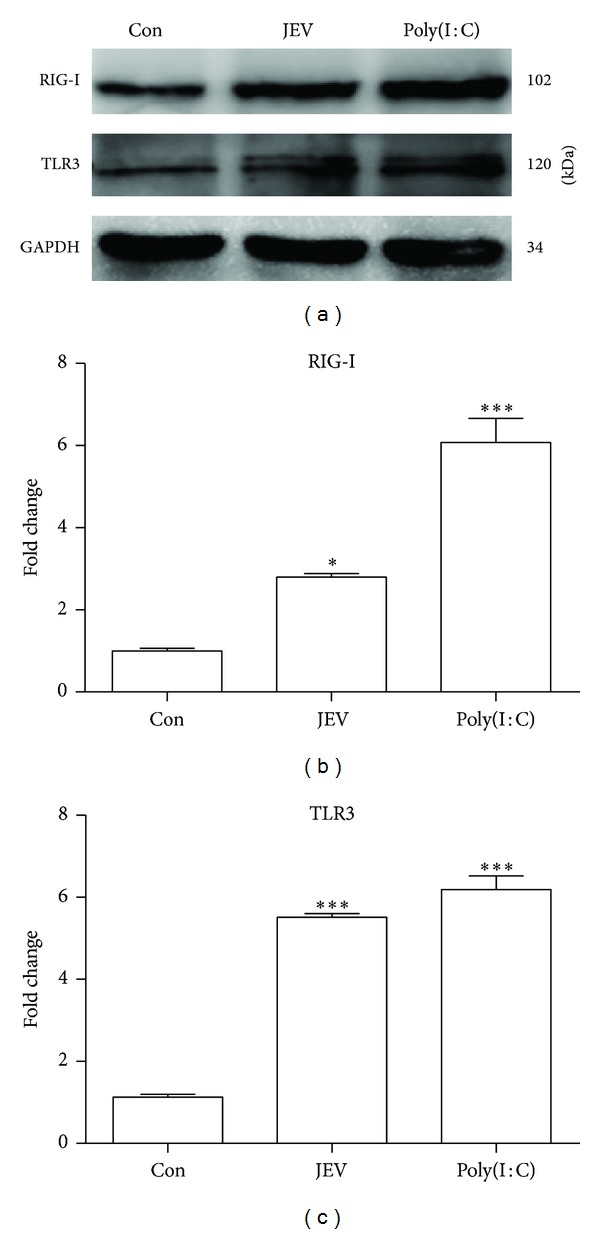
Expression of TLR3 and RIG-I in JEV-infected BV-2 cells. (a) BV-2 cells were either mock infected or infected with JEV at an MOI of 1. Poly(I : C) was added as a positive control. Western blotting was performed to detect TLR3 and RIG-I at 24 hpi. (b, c) The protein levels were quantified with immunoblot scanning and normalized to the amount of GAPDH. Error bars represent the standard deviation of results from three independent assays (*P < 0.05; ***P < 0.001).

Both RIG-1 and TLR3 have been recognized as important PRRs in viral infection, but their roles in JEV-infected microglia are not fully understood. To determine whether microglial cells use these PRRs for JEV recognition and induction of inflammatory mediators, the expression of RIG-I and TLR3 in JEV- or mock-infected BV-2 cells was analyzed with western blotting. The levels of both RIG-1 and TLR3 were markedly increased in JEV-infected and poly(I : C)-treated cells compared with controls (Figure 1), suggesting that JEV infection stimulates the expression of RIG-I and TLR3 in microglia.

### 3.2. JEV Activates ERK, p38MAPK, NF-*κ*B, and AP-1 in BV-2 Cells

**Figure 2:**
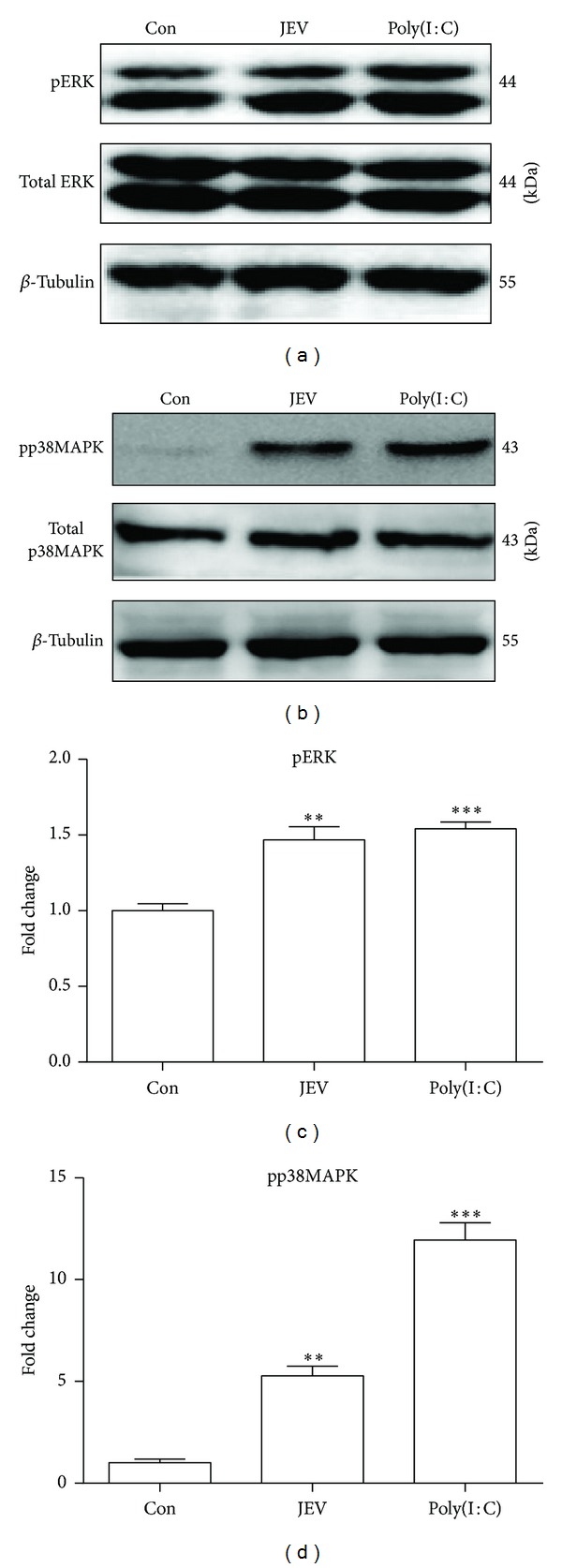
Activation of ERK and p38MAPK by JEV infection in BV-2 cells. BV-2 cells were either mock infected or infected with JEV at an MOI of 1. Poly(I : C) was added as a positive control. At 5 hpi, western blotting was performed to detect the phosphorylation of ERK (pERK) (a) and p38MAPK (pp38MAPK) (b). The protein levels were quantified with immunoblot scanning and normalized to the amount of *β*-tubulin (c and d). Error bars represent the standard deviation of results from three independent assays (**P < 0.01; ***P < 0.001).

**Figure 3:**
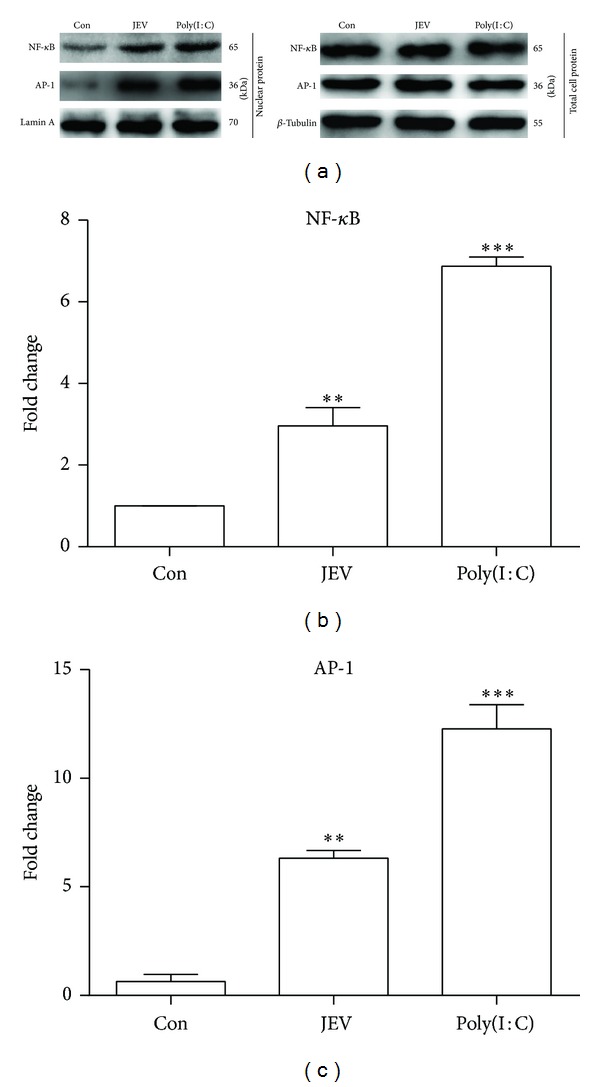
Nuclear localization of AP-1 and NF-*κ*B in JEV-infected BV-2 cells. BV-2 cells were either mock infected or infected with JEV at an MOI of 1. Poly(I : C) was added as a positive control. (a) AP-1 and NF-*κ*B in the nucleus and whole-cell extracts were detected with western blotting at 5 hpi. (b, c) These proteins were quantified with immunoblot scanning and normalized to the amount of lamin A or *β*-tubulin. The ratios of protein levels in the nucleus to levels in whole-cell extracts were calculated. Error bars represent the standard deviation of results from three independent assays (**P < 0.01; ***P < 0.001).

TLR and RIG-I-like receptor signaling activates transcription factors such as NF-*κ*B and AP-1, which induce production of proinflammatory cytokines [19]. p38MAPK and ERK are upstream activators of NF-*κ*B and AP-1 [16]. Thus, the status of these proteins was examined with western blotting. The levels of phosphorylated ERK and phosphorylated p38MAPK in JEV-infected cells were remarkably upregulated at 5 hpi compared with levels in control cells (*P* < 0.01) (Figure 2). NF-*κ*B and AP-1 levels were elevated in nuclear extracts of JEV-infected cells at 5 hpi (*P* < 0.01) (Figure 3). These results implied that JEV infection activates a signaling pathway involving ERK, p38MAPK, AP-1, and NF-*κ*B.

### 3.3. Knockdown of RIG-I and TLR3 Attenuates the Activation of p38MAPK, ERK, AP-1, and NF-*κ*B in JEV-Infected BV-2 Cells

**Figure 4:**
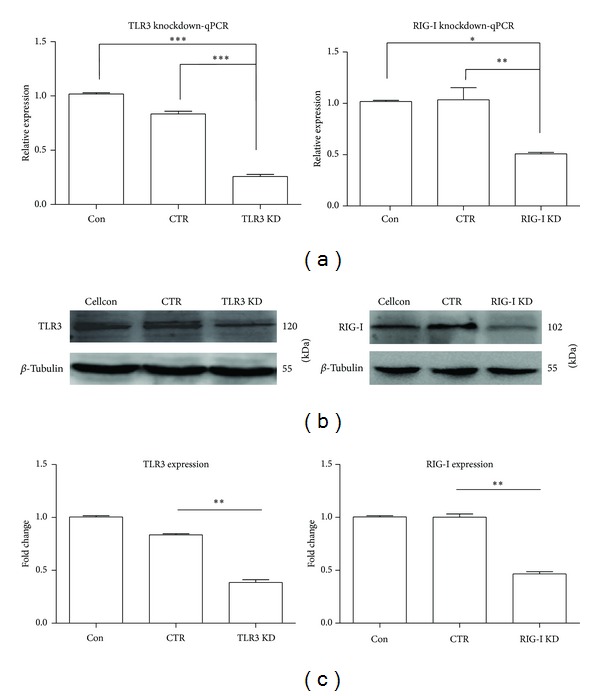
Knockdown of TLR3 and RIG-I in BV-2 cells with shRNA transfection. BV-2 cells were transfected with shRNA specific for TLR3 (TLR3 KD), RIG-I (RIG-I KD), or control (CTR). (a) At 48 h after transfection, total cellular RNA was isolated, and the levels of TLR3 and RIG-I mRNAs were determined with qRT-PCR. (b) Western blotting was performed to detect TLR3 and RIG-I. (c) Proteins were quantified with immunoblot scanning and normalized to the amount of *β*-tubulin. Error bars represent the standard deviation of results from three independent assays (*P < 0.05; **P < 0.01; ***P < 0.001).

**Figure 5:**
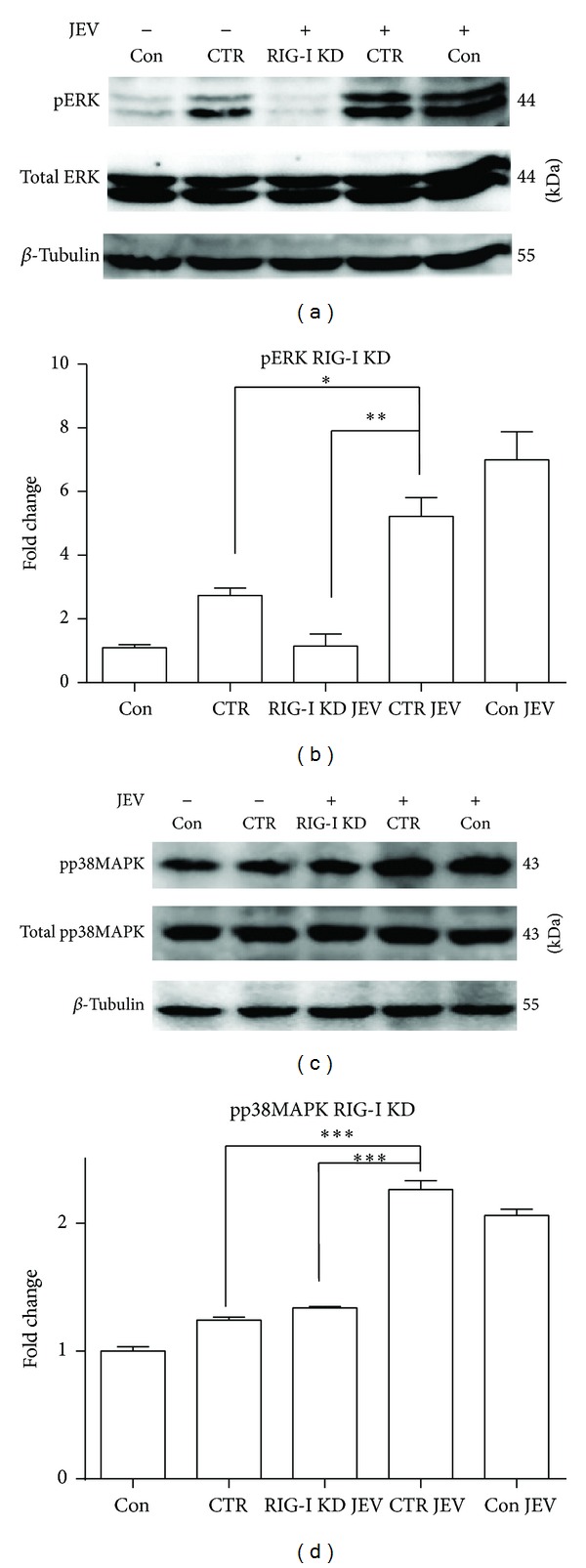
Knockdown of RIG-I attenuates the activation of p38MAPK and ERK in JEV-infected BV-2 cells. BV-2 cells were transfected with RIG-I shRNA (RIG-I KD) or negative control shRNA (CTR) and then infected with JEV at an MOI of 1. (a, c) Phospho-ERK (pERK) and phospho-p38MAPK (pp38MAPK) were detected with western blotting at 5 hpi. (b, d) The protein levels were quantified with immunoblot scanning and normalized to the amount of *β*-tubulin. Error bars represent the standard deviation of results from three independent assays (*P < 0.05; **P < 0.01; ***P < 0.001).

**Figure 6:**
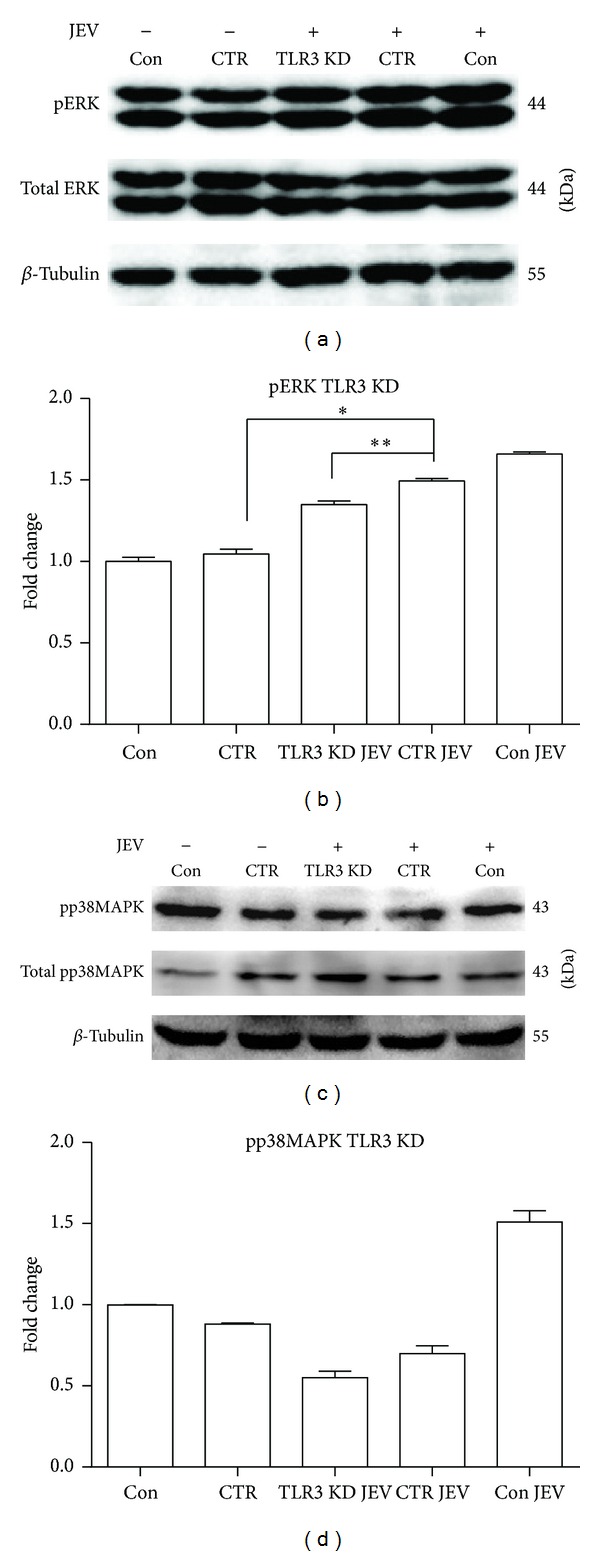
Knockdown of TLR3 decreases the activation of p38MAPK and ERK in JEV-infected BV-2 cells. BV-2 cells were transfected with TLR3 shRNA (TLR3 KD) or negative control shRNA (CTR) and then infected with JEV at an MOI of 1. (a, c) Phospho-ERK (pERK) and phospho-38MAPK (pp38MAPK) were detected with western blotting at 5 hpi. (b, d) The protein levels were quantified with immunoblot scanning and normalized to the amount of *β*-tubulin. Error bars represent the standard deviation of results from three independent assays (*P < 0.05; **P < 0.01).

**Figure 7:**
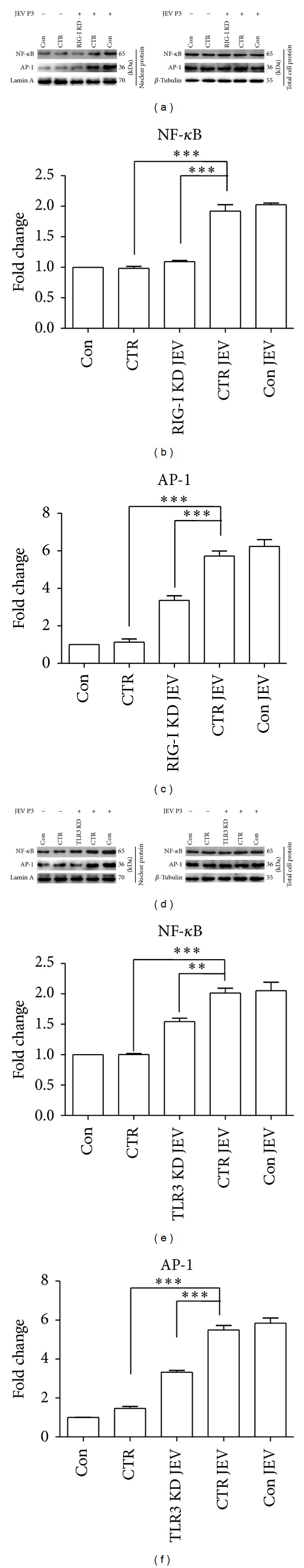
Knockdown of RIG-I and TLR3 reduces the nuclear translocation of AP-1 and NF-*κ*B. (a) BV-2 cells were transfected with RIG-I shRNA (RIG-I KD) or negative control shRNA (CTR) and then infected with JEV at an MOI of 1. AP-1 and NF-*κ*B in the nucleus were detected with western blotting at 5 hpi. (d) BV-2 cells were transfected with TLR3 shRNA (TLR3 KD) or negative control shRNA (CTR) and then infected with JEV at an MOI of 1. AP-1 and NF-*κ*B in the nucleus were detected with western blotting at 5 hpi. (b, c, e, f) The protein levels were quantified with immunoblot scanning and normalized to the amount of lamin A or *β*-tubulin. Error bars represent the standard deviation of results from three independent assays (**P < 0.01; ***P < 0.001).

To investigate the role of RIG-1 and TLR3 in the activation of NF-*κ*B and AP-1 signaling by JEV, shRNAs specifically targeting RIG-1 and TLR3 were transfected into BV-2 cells (Figure 4). Following RIG-I knockdown, the levels of phospho-ERK (*P* < 0.01), phospho-p38MAPK (*P* < 0.001) (Figure 5), and nuclear-localized AP-1 (*P* < 0.001) and NF-*κ*B (*P* < 0.001) in JEV-infected cells were significantly reduced compared with levels in JEV-infected cells treated with control shRNA (CTR) at 5 hpi (Figure 7). TLR3 knockdown also reduced the expression of phospho-ERK (*P* < 0.01) and nuclear-localized AP-1 (*P* < 0.001) and NF-*κ*B (*P* < 0.01) (Figures 6 and 7). However, the expression of phospho-p38MAPK was only slightly decreased upon TLR3 knockdown. These results indicated that RIG-I and TLR3 mediate the activation of signaling involving ERK, p38MAPK, AP-1, and NF-*κ*B and that RIG-I likely plays a more important role than TLR3.

### 3.4. Knockdown of RIG-I and TLR3 Reduces the Secretion of Proinflammatory Cytokines and Chemokines from JEV-Infected Microglia

**Figure 8:**
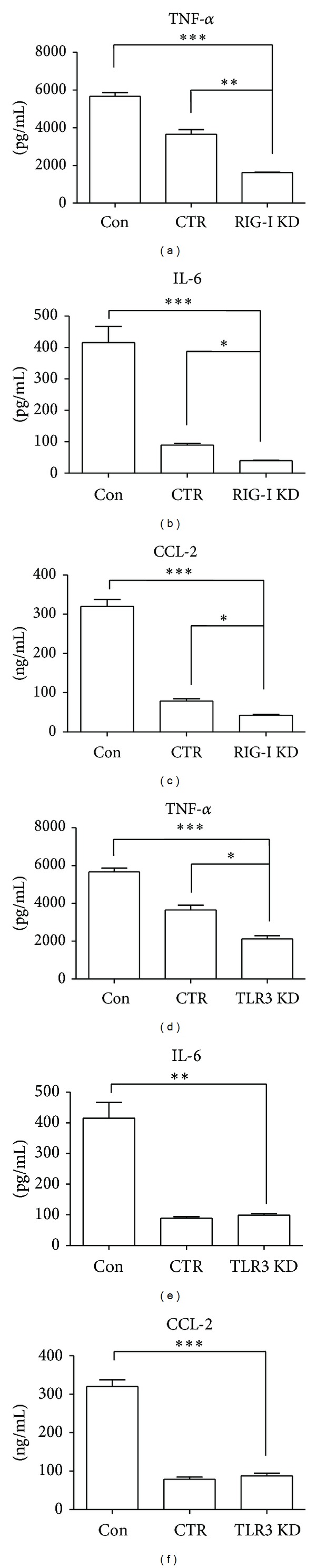
Proinflammatory cytokines and chemokines induced by JEV in TLR3 and RIG-I knockdown BV-2 cells. BV-2 cells were transfected with RIG-I shRNA (RIG-I KD), TLR3 shRNA (TLR3 KD), or negative control shRNA (CTR) and then infected with JEV at an MOI of 1. The production of TNF-*α* (a, d), IL-6 (b, e), and CCL-2 (c, f) was measured with ELISA. Error bars represent the standard deviation of results from three independent assays (*P < 0.05; **P < 0.01; ***P < 0.001).

We next investigated the importance of TLR3 and RIG-I in the expression of cytokines and chemokines, which were elevated upon JEV infection of BV-2 cells. The release of TNF-*α* (*P* < 0.01), IL-6 (*P* < 0.05), and CCL-2 (*P* < 0.05) was significantly reduced in the RIG-I knockdown cells compared with CTR cells (Figures 8(a)–8(c)). Interestingly, only TNF-*α* was reduced in TLR3 knockdown cells (*P* < 0.05), and we observed no obvious change in expression of IL-6 or CCL-2 (Figures 8(d)–8(f)). These results indicated that RIG-I likely plays a more critical role than TLR3 in activation of proinflammatory mediators in microglia following JEV infection.

### 3.5. Inhibitors of ERK and p38MAPK Suppress the Release of Proinflammatory Cytokines and Chemokines from JEV-Infected Microglia

**Figure 9:**
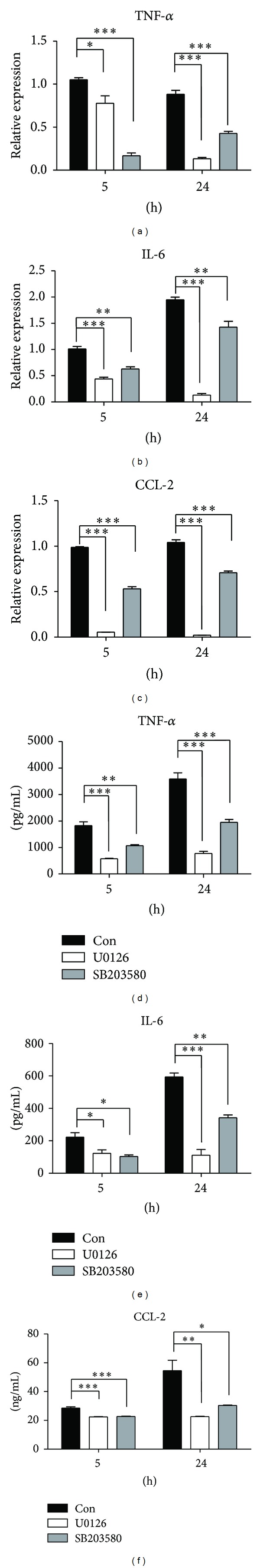
Inflammatory mediators induced by JEV in BV-2 cells treated with ERK and p38MAPK inhibitors. BV-2 cells were infected with JEV at an MOI of 1 and treated with an inhibitor of ERK (10 *μ*M U0126) or p38MAPK (10 *μ*M SB302580) after 1 h of incubation. The production of TNF-*α*, IL-6, and CCL-2 was measured with qRT-PCR (a–c) or ELISA (d–f) at 5 and 24 hpi. Error bars represent the standard deviation of results from three independent assays (*P < 0.05; **P < 0.01; ***P < 0.001).

The ERK inhibitor U0126 or the p38MAPK inhibitor SB302580 was added to JEV-infected cells, and the expression of proinflammatory cytokines and chemokines was detected with qRT-PCR and ELISA. Remarkably, a decrease in JEV-induced TNF-*α*, IL-6, and CCL-2 was observed in cells treated with these inhibitors (Figure 9), confirming that JEV-induced expression of proinflammatory cytokines and chemokines was mediated by the ERK and p38MAPK pathway.

### 3.6. Knockdown of RIG-I and TLR3 Increases the Viral Load in BV-2 Cells

**Figure 10:**
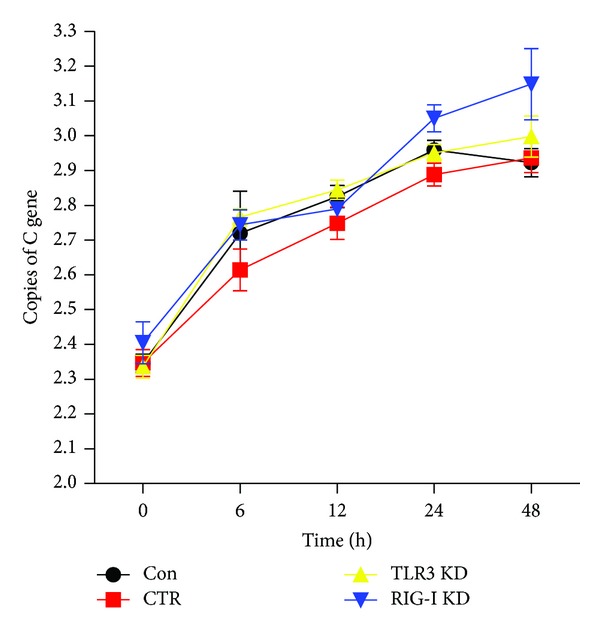
Viral load in TLR3 and RIG-I knockdown cells. BV-2 cells were transfected with RIG-I shRNA (RIG-I KD), TLR3 shRNA (TLR3 KD), or negative control shRNA (CTR) and then infected with JEV at an MOI of 0.01. Cells were collected at different time points, and total RNA was isolated. The number of copies of the JEV C gene was determined with qRT-PCR. Error bars represent the mean ± standard error from three independent experiments.

qRT-PCR was performed to detect viral replication in BV-2 cells that were transfected with shRNA targeting RIG-I or TLR3. We observed increased levels of JEV RNA in both RIG-I knockdown and TLR3 knockdown cells compared with levels in CTR cells. The number of copies of the C gene in RIG-I knockdown cells was greater than that in TLR3 knockdown cells at 24–48 hpi (Figure 10).

## 4. Discussion

Microglia play a key role in the proinflammatory response in the CNS and provide the first line of defense against invading microbes [20]. Previous studies showed a strong association between the presence of activated microglia and significant neuronal damage during JEV infection [7]. Neuronal death caused by JEV infection leads to astroglial and microglial activation and the release of proinflammatory mediators [18]. In addition, JEV-infected microglia express elevated levels of a number of proinflammatory mediators including IL-18, IL-1*β*, TNF-*α*, IL-6, and RANTES [16, 21, 22], which can induce neuron death. Thus, microglial cells may contribute to increased neuronal death via both infective and noninfective pathways.

dsRNA, a type of pathogen-associated molecular pattern, can be recognized by PRRs such as TLR3 and RIG-I. TLR3 is critical for inducing the cytokines, chemokines, and type I interferons in response to poly(I : C) treatment in microglia [12]. RIG-I is essential for the recognition of JEV that ultimately leads to the production of type I interferon in Vero (epithelial) cells, and RIG-I modulates the inflammatory response in JEV-infected neurons [8]. However, the role of TLR3 and RIG-I in JEV-induced inflammatory responses in microglia is unclear. Here we show the importance of the TLR3 and RIG-I signaling pathways in the JEV-induced inflammatory response in microglia. Recent studies have reported that microglia and astrocytes express numerous PRRs, which allow the recognition of diverse pathogen-associated molecular patterns. The direct demonstration that TLR3 regulates glial and/or neuronal responses to dsRNA prompts the examination of the response of primary cells isolated from TLR3-deficient mice. RIG-I is undetectable in uninfected neurons; in JEV-infected neurons, however, an upregulation of RIG-I and its downstream effectors IPS-1, TRAF6, FADD, and I*κ*B*α* as well as an increase in p38MAPK and NF-*κ*B phosphorylation was reported [6, 8]. Our study demonstrates that both TLR3 and RIG-I are detectable in normal BV-2 cells, and their levels are upregulated following JEV infection.

Induction of proinflammatory cytokines occurs primarily at the level of transcription initiation, through coordinate activation of several transcriptional factors, including NF-*κ*B and AP-1. Increasing evidence suggests that p38MAPK and ERK signaling cascades are involved in the activation of NF-*κ*B and AP-1. Our study showed that inhibition of ERK and p38MAPK activity effectively attenuated the expression of proinflammatory cytokines induced by JEV in BV-2 cells, indicating that JEV induces an inflammatory response via the ERK/p38MAPK pathway in microglia. This observation is partly consistent with a previous report which suggested that the Src/Ras/ERK signaling cascade is activated in JEV-infected neurons/glia [16]. Furthermore, significant reduction of phospho-ERK and phospho-p38MAPK was observed in JEV-infected cells after RIG-I knockdown (*P* < 0.01). When TLR3 was knocked down, however, only reduction of phospho-ERK was observed (*P* < 0.01; Figures 6(a) and 6(b)), suggesting that ERK but not p38MAPK was activated via the TLR3 pathway by JEV or that the effect of TLR3 knockdown was countered by activation of RIG-I signaling. Regarding the expression of the nuclear transcription factors AP-1 and NF-*κ*B, a greater difference was seen in RIG-I knockdown cells than in TLR3 knockdown cells (Figure 7). ELISA results showed that only the level of TNF-*α* was considerably reduced after TLR3 knockdown, and a decrease in TNF-*α*, IL-6, and CCL-2 expression was found following RIG-I knockdown (Figure 8). These observations indicate that RIG-I rather than TLR3 plays a leading role in modulating the levels of proinflammatory factors in microglia following JEV infection.

Interestingly, knockdown of RIG-I and TLR3 was associated with increased viral load in a proliferation assay (Figure 10), which was consistent with the results in RIG-I-knockdown neurons [8]. This phenomenon may be due to the fact that the antiviral innate immune response is subverted following RIG-I ablation. The effect of RIG-I knockdown on viral replication was more obvious than knockdown of TLR3, which further suggests that RIG-I plays a more important role than TLR3 in restricting JEV replication. In the case of West Nile virus (WNV) infection, however, TLR3 plays a protective role against infection of neurons with this virus [23]. These differences may be due to different cell types and viruses.

In conclusion, our research demonstrates that sensing of JEV via TLR3 and RIG-I leads to modulation of the ERK/p38MAPK and AP-1/NF-*κ*B pathways, which mediate the participation of microglia in the inflammatory response in the CNS. These results enrich our understanding of JEV pathogenesis.

## Figures and Tables

**Table 1:** Primers sequences used in qPCR.

Name	Forward primer	Reverse primer
*β*-Actin	5′-CACTGCCGCATCCTCTTCCTCCC-3′	5′-CAATAGTGATGACCTGGCCGT-3′
TNF-*α*	5′-TGTCTCAGCCTCTTCTCATTCC-3′	5′-TTAGCCCACTTCTTTCCCTCAC-3′
CCL-2	5′-CGGCGAGATCAGAACCTACAAC-3′	5′-GGCACTGTCACACTGGTCACTC-3′
IL-6	5′-CATGTTCTCTGGGAAATCGTG-3′	5′-TCCAGTTTGGTAGCATCCATC-3′
C gene	5′-GGCTCTTATCACGTTCTTCAAGTTT-3′	5′-TGCTTTCCATCGGCCYAAAA-3′

## References

[1] Unni SK, Růžek D, Chhatbar C, Mishra R, Johri MK, Singh SK (2011). Japanese encephalitis virus: from genome to infectome. *Microbes and Infection*.

[2] Ghosh D, Basu A (2009). Japanese encephalitis—a pathological and clinical perspective. *PLoS Neglected Tropical Diseases*.

[3] Gupta N, Santhosh SR, Babu JP, Parida MM, Rao PVL (2010). Chemokine profiling of Japanese encephalitis virus-infected mouse neuroblastoma cells by microarray and real-time RT-PCR: implication in neuropathogenesis. *Virus Research*.

[4] Mackenzie JS, Gubler DJ, Petersen LR (2004). Emerging flaviviruses: the spread and resurgence of Japanese encephalitis, West Nile and dengue viruses. *Nature Medicine*.

[5] Aloisi F (2001). Immune function of microglia. *Glia*.

[6] Olson JK, Miller SD (2004). Microglia initiate central nervous system innate and adaptive immune responses through multiple TLRs. *Journal of Immunology*.

[7] Ghoshal A, Das S, Ghosh S (2007). Proinflammatory mediators released by activated microglia induces neuronal death in Japanese encephalitis. *GLIA*.

[8] Nazmi A, Dutta K, Basu A (2011). RIG-I mediates innate immune response in mouse neurons following Japanese encephalitis virus infection. *PLoS ONE*.

[9] Savarin C, Bergmann CC (2008). Neuroimmunology of central nervous system viral infections: the cells, molecules and mechanisms involved. *Current Opinion in Pharmacology*.

[10] Alexopoulou L, Holt AC, Medzhitov R, Flavell RA (2001). Recognition of double-stranded RNA and activation of NF-*κ*B by toll-like receptor 3. *Nature*.

[11] Yu M, Levine SJ (2011). Toll-like receptor 3, RIG-I-like receptors and the NLRP3 inflammasome: key modulators of innate immune responses to double-stranded RNA viruses. *Cytokine and Growth Factor Reviews*.

[12] Town T, Jeng D, Alexopoulou L, Tan J, Flavell RA (2006). Microglia recognize double-stranded RNA via TLR3. *Journal of Immunology*.

[13] Suh H, Brosnan CF, Lee SC (2009). Toll-like receptors in CNS viral infections. *Current Topics in Microbiology and Immunology*.

[14] Mikkelsen SS, Jensen SB, Chiliveru S (2009). RIG-I-mediated activation of p38 MAPK is essential for viral induction of interferon and activation of dendritic cells. Dependence on TRAF2 and TAK1. *The Journal of Biological Chemistry*.

[15] Wu YP, Chang CM, Hung CY, Tsai MC, Schuyler SC, Wang RY (2011). Japanese encephalitis virus co-opts the ER-stress response protein GRP78 for viral infectivity. *Virology Journal*.

[16] Chen C, Ou Y, Chang C (2011). Src signaling involvement in Japanese encephalitis virus-induced cytokine production in microglia. *Neurochemistry International*.

[17] Petrai I, Rombouts K, Lasagni L (2008). Activation of p38MAPK mediates the angiostatic effect of the chemokine receptor CXCR3-B. *International Journal of Biochemistry and Cell Biology*.

[18] Swarup V, Ghosh J, Das S, Basu A (2008). Tumor necrosis factor receptor-associated death domain mediated neuronal death contributes to the glial activation and subsequent neuroinflammation in Japanese encephalitis. *Neurochemistry International*.

[19] Sánchez-Zauco NA, Giono-Cerezo S, Maldonado-Bernal C (2010). Toll-like receptors, pathogenesis and immune response to *Helicobacter pylori*. *Salud Publica de Mexico*.

[20] Lehnardt S (2010). Innate immunity and neuroinflammation in the CNS: the role of microglia in toll-like receptor-mediated neuronal injury. *Glia*.

[21] Das S, Mishra MK, Ghosh J, Basu A (2008). Japanese Encephalitis Virus infection induces IL-18 and IL-1*β* in microglia and astrocytes: Correlation with in vitro cytokine responsiveness of glial cells and subsequent neuronal death. *Journal of Neuroimmunology*.

[22] Chen C, Ou Y, Lin S (2010). Glial activation involvement in neuronal death by Japanese encephalitis virus infection. *Journal of General Virology*.

[23] Daffis S, Samuel MA, Suthar MS, Gale M, Diamond MS (2008). Toll-like receptor 3 has a protective role against West Nile virus infection. *Journal of Virology*.

